# Effect of BAFF blockade on the B cell receptor repertoire and transcriptome in a mouse model of systemic lupus erythematosus

**DOI:** 10.3389/fimmu.2023.1307392

**Published:** 2024-01-09

**Authors:** Tao Huang, Chenyu Pi, Xiaoqing Xu, Yan Feng, Jingming Zhang, Hua Gu, Jianmin Fang

**Affiliations:** ^1^ School of Life Sciences and Technology, Tongji University, Shanghai, China; ^2^ Biomedical Research Center, Tongji University Suzhou Institute, Suzhou, Jiangsu, China; ^3^ Department of Neurology, Tongji Hospital, Tongji University, Shanghai, China

**Keywords:** systemic lupus erythematosus, B cell receptor (BCR) repertoire, transcriptome, B cell-activating factor (BAFF), MRL/lpr

## Abstract

**Introduction:**

Systemic lupus erythematosus (SLE) is a heterogeneous autoimmune disease. Anti-B-cell-activating factor (BAFF) therapy effectively depletes B cells and reduces SLE disease activity. This research aimed to evaluate the effect of BAFF blockade on B cell receptor (BCR) repertoire and gene expression.

**Methods:**

Through next-generation sequencing, we analyzed gene expression and BCR repertoire in MRL/lpr mice that received long-term anti-BAFF therapy. Based on gene expression profiles, we predicted the relative proportion of immune cells using ImmuCellAI-mouse, validating our predictions via flow cytometry and FluoroSpot.

**Results:**

The loss of BCR repertoire diversity and richness, along with increased clonality and differential frequency distribution of the immunoglobulin heavy chain variable (IGHV) segment gene usage, were observed in BAFF-blockade mice. Meanwhile, the distribution of complementarity-determining region 3 (CDR3) length and CDR3 amino acid usage remained unaffected. BAFF blockade resulted in extensive changes in gene expression, particularly that of genes related to B cells and immunoglobulins. Besides, the tumor necrosis factor (TNF)-α responses and interferon (IFN)-α/γ were downregulated, consistent with the decrease in IFN-γ and TNF-α serum levels following anti-BAFF therapy. In addition, BAFF blockade significantly reduced B cell subpopulations and plasmacytoid dendritic cells, and caused the depletion of antibody-secreting cells.

**Discussion:**

Our comparative BCR repertoire and transcriptome analyses of MRL/lpr mice subjected to BAFF blockade provide innovative insights into the molecular pathophysiology of SLE.

## 1 Introduction

Systemic lupus erythematosus (SLE) is a multisystem autoimmune disease characterized by abnormal immune activity that causes tissue damage and extensive inflammation in multiple organs (1). Disruption of immune tolerance mechanisms leads to the massive proliferation and activation of B and T cells, which in turn produce autoantibodies and proinflammatory cytokines (2). B-cell-activating factor (BAFF, BLyS), which belongs to the tumor necrosis factor (TNF) ligand family, plays a key role in B-cell proliferation, differentiation, and activation (3). Elevated serum levels of BAFF correlate positively with anti-double-stranded DNA antibody (anti-dsDNA) titers and an increased present-day risk of autoimmunity (4, 5). To date, belimumab and telitacicept are the only two biologics against BAFF that have been approved for SLE therapy worldwide (6, 7).

Considering the central role of B cells in SLE pathogenesis, several researchers have explored changes in the B cell receptor (BCR) repertoire. Tipton and Odendah reported that the plasma cells of SLE patients exhibited increased usage of the immunoglobulin heavy chain variable (IGHV) 4 family, especially IGHV4-34, which encodes 9G4+ antibodies that target double-stranded DNA (8, 9). More diverse complementarity-determining region 3 (CDR3) sequences have been observed in the B cell receptor (BCR) repertoire of patients with SLE than in healthy controls (10). Additionally, a significant shortening of CDR3 length in peripheral blood BCR has been reported in the former (11).

Transcriptome analyses of whole blood samples have been used to identify the biomarkers and pathogenic drivers of SLE. The expression of interferon (IFN)-stimulated genes (ISGs), more specifically type I IFN-stimulated genes, has been reported to correlate with SLE disease activity (12, 13). High ISG expression leads to autoantibody production together with the abnormal proliferation of B cells (14). A whole-blood transcriptome study of 158 pediatric patients with SLE found an enrichment of plasmablast and neutrophil transcripts (15). Based on gene expression profiles, Akhtar et al. estimated immune cell proportions using CIBERSORTx and found that patients with SLE had more naïve CD4 T cells and fewer memory-activated CD4 T cells than patients exposed to mycophenolate mofetil (16).

The complex interplay between BAFF, BAFF receptor, and BCR, can reshape the BCR repertoire and result in a skewed usage of the IGHV gene (17–19).. However, changes in the BCR repertoire and transcriptome of SLE murine models treated with biologics against BAFF remain largely unknown. MRL/lpr mice develop an autoimmune disease resembling SLE caused by a mutation in the Fas gene, which promotes the survival of self-reactive lymphocytes (20). In this study, we performed a thorough analysis of the BCR repertoire and transcriptome of MRL/lpr mice treated with the BAFF receptor fusion protein (BAFF-R-Fc). Our results demonstrate that the frequency distribution of the IGHV gene usage was distinct from those of controls. We also observed a decrease in BCR diversity and richness, and an increase in clonality in the treatment group concurrently. Furthermore, blocking BAFF downregulates genes related to B cell proliferation and antibody production, and alters the composition of splenic immune cells, especially by reducing the number of B cell subsets. Overall, our findings advance a better knowledge of the mechanisms of BAFF blockade against SLE.

## 2 Methods

### 2.1 Mice and mouse BAFF-R-Fc fusion protein

Female 6-week-old MRL/lpr mice were purchased from Shanghai Laboratory Animal Co., Ltd. (Shanghai, China). All mice were raised under specific pathogen-free conditions. Mouse BAFF-R-Fc fusion protein was provided by RemeGen Co., Ltd. (Yantai, China). All experimental procedures were performed following the regulations of the Animal Ethics Committee of Tongji University (No. TJLAC-018-032).

### 2.2 Animal experiment

The treatment group received BAFF-R-Fc at a dose of 10 mg/kg, whereas the control group received normal saline at the age of 8 weeks. All drugs were injected subcutaneously once per day for a total of 28 injections.

### 2.3 BCR repertoire construction, sequencing, and analysis

Total RNA was extracted from the MRL/lpr mice spleens after treatment using Trizol according to the user manual. 5’RACE was performed for reverse-transcription, and 5’RACE template switch oligos contain unique molecular identifiers (HiScript-TS 5’/3’ RACE Kit, Vazyme, China). Reverse transcription products were amplified via PCR using specific primers designed for IgG and IgM sequences (primers were provided by Azenta Life Sciences, Chelmsford, MA, USA). PCR products were purified by DNA magnetic beads. PCR purified product is terminal repair (including 5 ‘terminal phosphorylation and 3’ terminal plus’ A ‘) by the End Prep Enzyme Mix, with sequencing adapters at both ends. Then PCR products were amplified with P5 and P7 primers, cleaned up using beads, validated using a Qsep100 (Bioptic, Taiwan, China), and quantified by Qubit3.0 Fluorometer (Invitrogen, Carlsbad, CA, USA). Sequencing was performed with an Illumina MiSeq instrument and carried out using a 2×300 paired-end configuration, according to the manufacturer’s instructions (Illumina, San Diego, CA, USA).

Raw fastq files were first subject to quality assessment. Adapters and bases with poor quality scores (Q value lower than 20) were removed using Cutadapt (V1.9.1) to generate clean data. Paired-end reads were merged using FLASH (version 2.2.00). The merged sequences were BLASTed against the IMGT reference database to identify the best match between germline V(D)J genes and sequences in the CDR1, CDR2, and CDR3 regions using MIXCR (V3.0.13). To assess BCR repertoire diversity, IGHV, and IGHJ gene usage, distributions of CDR3 region length, and clone information, clonotype sequences were analyzed using the immunarch R package (21).

### 2.4 RNA-seq and data analysis

RNA-seq was performed using 1 μg total RNA (Azenta Life Sciences, Chelmsford, MA, USA). The poly(A) mRNA isolation was performed using Oligo(dT) beads. The mRNA fragmentation was performed using divalent cations and high temperature. Priming was performed using Random Primers. First strand cDNA and the second-strand cDNA were synthesized. The purified double-stranded cDNA was then treated to repair both ends and add a dA-tailing in one reaction, followed by a T-A ligation to add adaptors to both ends. Size selection of Adaptor-ligated DNA was then performed using DNA Clean Beads. Each sample was then amplified by PCR using P5 and P7 primers and the PCR products were validated. Sequencing was performed on an Illumina HiSeq instrument and carried out using a 2×150 paired-end configuration, according to the manufacturer’s instructions (Illumina, San Diego, CA, USA).

The DESeq2 Bioconductor package was performed to identify differentially expressed genes (DEGs) (22, 23), which were defined as those with an adjusted *p*-value < 0.05 and |log2 fold change| ≥ 1. ClusterProfiler and pathview R packages were used to identify Kyoto Encyclopedia of Genes and Genomes (KEGG) pathway terms and Gene Ontology (GO) biological process, with a significant adjusted *p*-value < 0.05 as the threshold (24). Gene set enrichment analysis (GSEA) was used to identify differentially enriched hallmark gene sets downloaded from the MSigDB database using the ClusterProfiler R package. *p*-value < 0.05 and |Normalized enrichment score| > 1 were set as the cutoffs.

Immune-related DEGs were submitted to the STRING database to obtain interaction information, with the cutoff interaction score set at 0.7. The visualization of the protein-protein interaction (PPI) network was performed using Cytoscape software. The betweenness centrality for each gene was estimated using the cytoNCA plugin of Cytoscape. DEGs were arranged according to the value of betweenness centrality. The ImmuCellAI-mouse online tool was used to predict the relative proportion of immune cells based on RNA-seq data (25). The TRRUST v2 database, which contains 6552 transcription factors (TFs)-target interactions between mouse TFs, was used to identify key TFs among DEGs (26).

### 2.5 Measurement of immunoglobulin isotype, anti-dsDNA antibody titer, and immunoregulatory cytokines levels in serum

Anti-dsDNA antibody titers were measured using an ELISA kit (Alpha Diagnostic Intl. Inc. Texas, USA). Immunoglobulin (IgA, IgM, IgG1, IgG2a, IgG2b, IgG3) and cytokine (IL-10, IFN-γ, TNF-α) levels in serum were measured with a LEGENDplex™ CBA kit (BioLegend, San Diego, CA, USA), using a CytoFLEX LX (Beckman Coulter, Brea, CA, USA), according to the manufacturer’s instructions.

### 2.6 Analysis of B cell subsets in the spleen via flow cytometry

MRL/lpr mice were sacrificed on the day after the 28th treatment to obtain the spleens. Forty-µm mesh filters (BioFIL, Shanghai, China) were used to obtain single-cell suspensions in phosphate-buffered saline (BioFIL, Shanghai, China). The sorting strategy for splenic lymphocytes was based on OMIP 076 (27). Subsets of B cell lymphocytes were analyzed using CytoFLEX LX (Beckman Coulter, Brea, CA, USA) after incubation with the following antibody combinations: PerCP/Cyanine5.5 anti-mouse CD45R/B220, TruStain FcX™ (anti-mouse CD16/32) Antibody, APC/Cyanine7 anti-mouse CD19, FITC anti-mouse TCRβ chain, PE/Cyanine7 anti-mouse CD138 (Syndecan-1), APC anti-mouse IgM, Brilliant Violet 785™ anti-mouse I-A/I-E, APC anti-mouse CD1d (CD1.1, Ly-38), Zombie Aqua™, PE anti-mouse CD5, PE anti-mouse PD-L2. All antibodies were purchased from BioLegend (San Diego, CA, USA).

### 2.7 Detection of antibody-secreting cells in the spleen via FluoroSpot assay

The number of IgM, IgA, and IgG ASCs was determined using FluoroSpot flex (MABTECH AB, Sweden) following the manufacturer’s instructions. In brief, 5×10^4^ cells were added per well in 200 µl cell culture medium containing 10% fetal bovine serum. The cells were then incubated overnight in a 37°C humidified incubator with 5% CO2. Cells were then removed and detection antibodies were added to per well and incubated for 2 hours at 20°C. The spots were counted using a FluoroSpot reader (MABTECH AB, Sweden).

### 2.8 Statistical analysis

The statistical significance of difference between the two groups was analyzed using the unpaired two-tailed t-test or Mann-Whitney U-test. P-value adjusting is done using the Holm-Bonferroni correction in multiple testing. Pearson correlation coefficient was performed to indicate the extent of linear correlation between two arbitrary variables. Statistical analysis was performed using GraphPad Prism version 9.5 and R software version 4.3.0. Data were presented as the mean ± standard deviation. A p-value < 0.05 was regarded as statistically significant. *p < 0.05; **p < 0.01; ***p < 0.001.

## 3 Results

### 3.1 Changes in BCR repertoire after BAFF blockade

**Figure 1:**
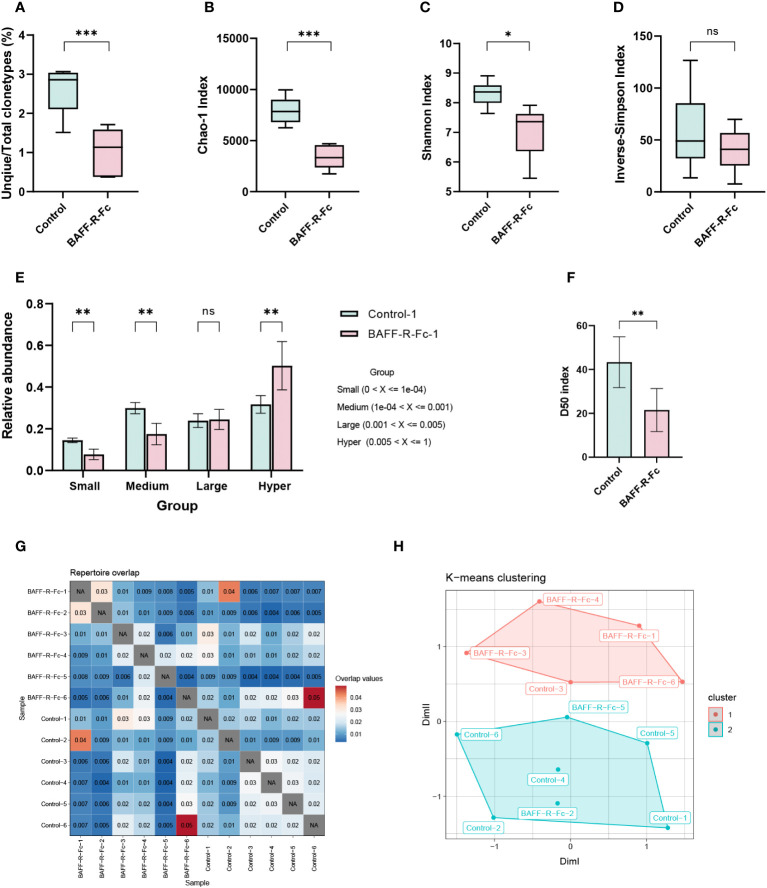
Analysis of BCR repertoire in the spleen of MRL/lpr mice. **(A)** The number of unique clonotypes. **(B)** Clonotypes richness estimated by Chao1 index. **(C)** Quantification of the BCR repertoire diversity using the Shannon index and **(D)** the inverse-Simpson index. **(E)** The cumulative relative abundance of small-expanded (0-0.01%), medium-expanded (0.01%-0.1%), large-expanded (0.1%-0.5%), and hyper-expanded (0.5%-100%) clonotypes in two groups (mean ± S.D, Mann-Whitney U-test and Holm-Bonferroni correction). **(F)** The bar plot of the D50 diversity index, D50 represents the number of clonotypes occupying 50% of repertoires. **(G)** Visualization of overlap coefficient among samples to measure repertoire similarity. **(H)** Multi-dimensional scaling (MDS) is applied to overlap coefficient, then clusters the MDS resulting components using K-means. Statistical analysis was performed with unpaired t-test comparing untreated to treated. Bars represent mean ± S.D. *p < 0.05; **p < 0.01; ***p < 0.001: ns, not significant.

To analyze BCR repertoire clonotypes in MRL/lpr mice, we performed next-generation sequencing of the BCR transcripts expressed by splenic B cells. First, we determined the ratio of unique/total BCR sequences. Notably, the ratio of unique/total BCR clonotypes in the treatment group was significantly lower than in the control group (**Figure 1A**). Next, we used ecological parameters to quantitatively measure repertoire diversity and complexity (28). The Chao1 index is a non-parametric asymptotic estimator of clonotype richness. Compared with the control, the treated MRL/lpr mice had a lower Chao1 index for the BCR repertoire (**Figure 1B**). To measure repertoire diversity, we used the Shannon index. A higher Shannon index indicates greater diversity of the BCR repertoire. A significant reduction in the Shannon index was noted compared to the control (**Figure 1C**). We also evaluated the inverse Simpson’s index for a more precise assessment. Consistent with the Shannon index results, the inverse Simpson’s index was reduced in the treatment group, although no statistical difference was observed (**Figure 1D**). Collectively, after BAFF-R-Fc treatment, the BCR repertoire of MRL/lpr mice exhibited a lower ratio of unique/total clonotypes, together with an obvious reduction in richness and diversity.

According to the clonotype proportions in total sequences (**Supplementary Figure 1A**), we classified these into four groups to assess the clonal space homeostasis: small-expanded (0-0.01%), medium-expanded (0.01%-0.1%), and large-expanded (0.1%-0.5%) and hyper-expanded (0.5%-100%). The cumulative relative abundances of these clonotypes were estimated (**Figure 1E**; **Supplementary Figure 1B**), as shown, the proportion of hyper-expanded clonotypes in the treatment group was significantly higher than that in the control, whereas the proportions of small- and medium-expanded clonotypes were significantly reduced. We also considered the most abundant cell clonotypes. Top 11-100 (*p* = 0.011) clonotypes occupied more repertoire space in the treatment group than those in the control, while 1001-3000 (*p* = 0.011), and 3001-10000 (*p* = 0.011) clonotypes occupied less repertoire space (**Supplementary Figures 1C, D**). Additionally, we estimated the relative proportion of the least prolific clonotypes. The proportions of clonotypes count 1, 2-3, 4-10, 11-30, and 31-100 were significantly lower in the treatment group than those in control, while clonotypes count more than 100 occupied more proportion (**Supplementary Figures 1E, F**). The diversity 50 index represents the number of clonotypes that occupy 50% of all clonotypes observed in the BCR repertoire. We found that the diversity 50 index in the treatment group was markedly less than that in the control group (**Figure 1F**). In conclusion, these outcomes indicate that fewer clonotypes tended to expand massively in treated mice.

To measure repertoire similarity, we took advantage of repertoire overlap by computing the overlap coefficient, which was defined as the ratio of the intersection’s size to the smaller size of the two sets. (**Figure 1G**). With this result, multidimensional scaling (MDS) clustered using K-means was applied to visualize repertoire similarity. MDS partially segregated the treated mice from the control group, as four of six samples from the treated group were highly clustered (**Figure 1H**). This distinction existed in the repertoire of drug-treated and control MRL/lpr mice.

### 3.2 Comparison of IGHV and immunoglobulin heavy chain joining gene usage

**Figure 2:**
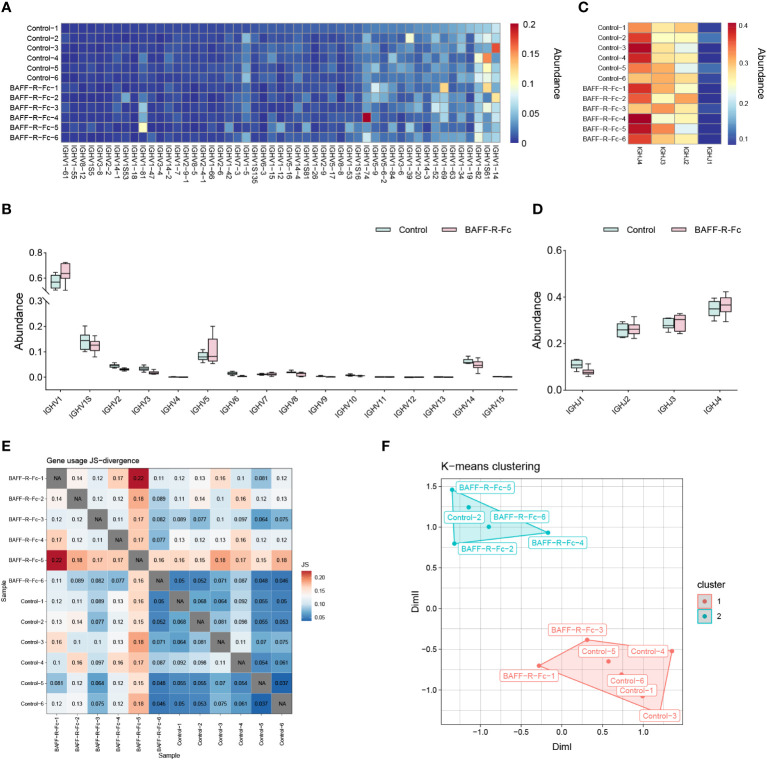
Differential usage of IGHV/J genes in BCR repertoires of MRL/lpr mice. **(A)** Heat map of IGHV gene usage showing those abundances > 0.1% among IGH sequences from each sample. The color indicates clone abundance per sample, as indicated in the legend. **(B)** The frequency of IGHV gene families in the BAFF-R-Fc treated and control group. **(C)** Heat map of IGHJ gene usage of each sample. **(D)** IGHJ gene usage is shown in the boxplot. **(E)** Visualization of Jensen-Shannon divergence between samples for IGHV genes usage analysis. **(F)** Principal components analyses (PCA) of Jensen-Shannon divergence, then clusters the PCA resulting components using K-means. Statistical analysis was performed with Mann-Whitney U-test and Holm-Bonferroni correction comparing untreated to treated. Bars represent mean ± S.D.

To determine whether BAFF blockade alters the usage of IGHV and IGHJ genes, we calculated the proportion of V and J alleles. The IGHV genes with frequency > 0.1% are shown in **Figure 2A**. IGHV1-14, IGHV1S61, and IGHV1-82 showed extremely high abundance (> 5%) in both groups (**Supplementary Table 1**). We compared the proportion of IGHV allele usage between the two groups, and found no significant difference in VH gene usage between them (**Supplementary Table 1**). We then analyzed the frequency of IGHV gene families in two groups. IGHV1 family was the most commonly used gene family in both groups (**Figure 2B**). And the frequency of IGHV1 family in BAFF-R-fc group did not reach statistical significance (64% ± 8%) compared to the control (57% ± 5%). The frequencies of IGHJ alleles are shown in **Figure 2C**. No statistical difference was observed in the usage of IGHJ alleles, and IGHJ4 was the most commonly used gene in both groups (**Figure 2D**). To fully understand the differences in gene usage, we estimated the Jensen-Shannon divergence between the two samples (**Figure 2E**), a method of measuring similarity between two probability distributions. A higher Jensen-Shannon divergence indicated a larger difference in the distribution of IGHV gene usage. Based on this result, PCA clustered using K-means was applied to map IGHV gene usage. As shown in **Figure 2F**, the PCA significantly segregated four samples in the treatment group from the control group, whereas two samples could not be distinguished. Together, these results suggest that the usage of IGHV alleles in treated mice differed from that in control mice.

### 3.3 Characteristics of CDR3 sequences

**Figure 3:**
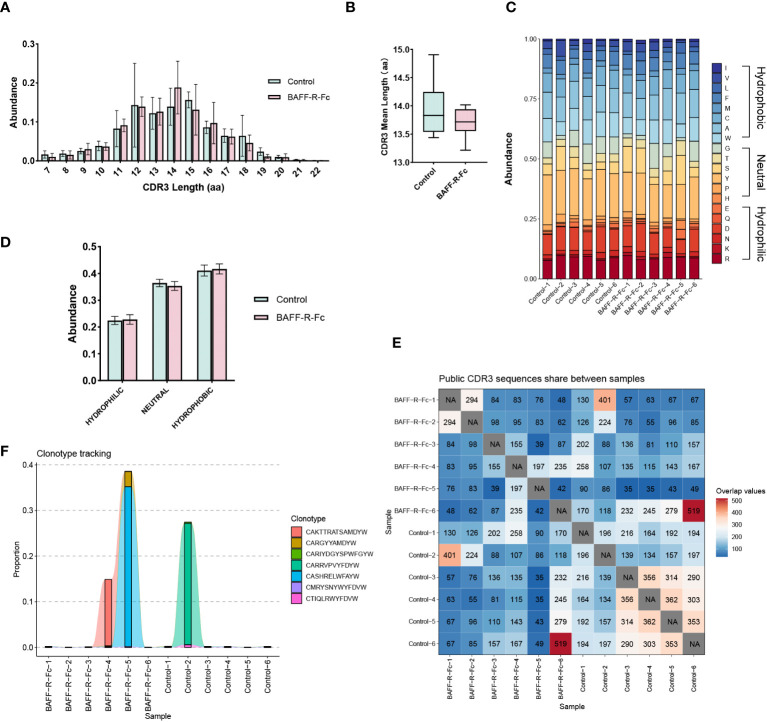
Characteristics of the CDR3 sequences in BCR repertoires of MRL/lpr mice. **(A)** Distribution of the length of CDR3 region in treated and control groups. **(B)** CDR3 region mean length in two groups (mean ± S.D., unpaired t-test). **(C)** Stacked plot displaying CDR3 region amino acid composition in each sample. **(D)** The usage of hydrophilic, neutral, and hydrophobic amino acids in two groups. **(E)** Heatmap of public CDR3 sequences shared between samples. **(F)** Tracking the seven CDR3 amino acid sequences shared between most samples. Statistical analysis was performed with Mann-Whitney U-test and Holm-Bonferroni correction comparing untreated to treated. Bars represent mean ± S.D.

CDR3 region of the immunoglobulin heavy chain is a key region for recognizing and binding antigens (29). Therefore, we analyzed the distribution of CDR3 length. As shown in **Figure 3A**, the most common CDR3 length in the control mice was 15 amino acids. In BAFF-R-Fc-treated mice, the most common CDR3 length decreased to 14 amino acids. However, no statistical differences in the usage of amino acids with different lengths were observed. We investigated the mean length of CDR3 sequences. The CDR3 mean length observed in BAFF-R-Fc-treated mice (13.71± 0.27) did not reach statistical significance compared to the control (13.93 ± 0.52) (**Figure 3B**). We also estimated the 20-amino-acid composition of CDR3 (**Figure 3C**). We then determined the cumulative relative abundances of hydrophilic, neutral, and hydrophobic amino acids. No statistically significant differences were observed in the usage of these three amino acid types (**Figure 3D**). We identified the public CDR3 sequences of BCR heavy chains shared between two samples and exhibited in **Figure 3E**, these shared CDR3 sequences had different unique molecular identifiers. For all samples, only three CDR3 sequences, CAKTTRATSAMDYW, CARRVPVYFDYW and CASHRELWFAYW, were used in all samples (**Supplementary Table 2**). And we tracked seven CDR3 sequences shared between most samples (**Figure 3F**). Noticeably, a few CDR3 sequences exhibited extremely high proportions in a specific sample.

### 3.4 Distinct transcriptional signatures linked to BAFF-R-Fc treatment responses

**Figure 4:**
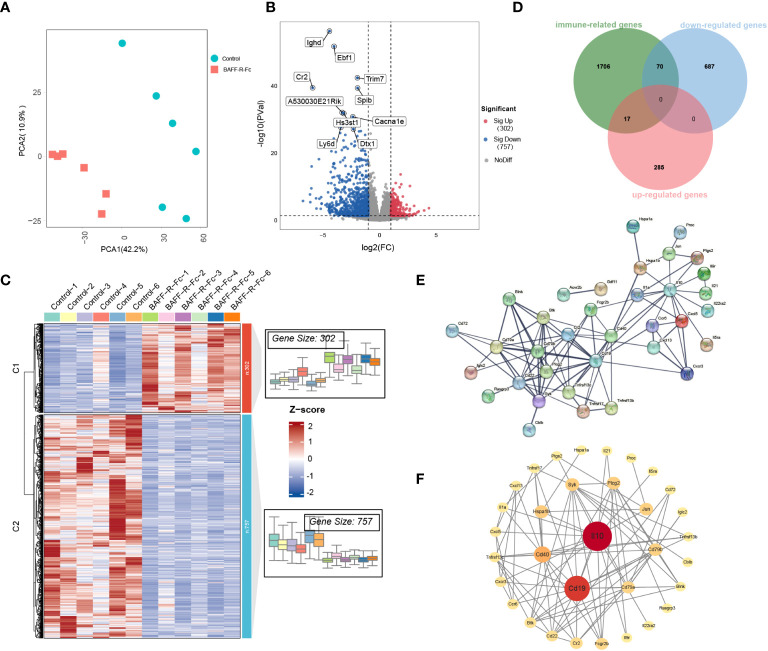
Distinct transcriptional signatures in the spleen of MRL/lpr mice received BAFF-R-Fc. **(A)** Principal components analyses (PCA) of spleen gene expression profiles from BAFF-R-Fc treated group and control. The first two principal components PCA1 and PCA2 are evaluated for each sample vector and plotted. **(B)** Volcano plot and **(C)** heatmap of differential expression genes between treatment and control group. Red points denote upregulated genes, blue points denote downregulated genes, and gray points denote genes with no significant differences. **(D)** Venn diagram of the overlap among immune-related genes (IRGs) and upregulated/downregulated DEGs. IRGs are obtained from the Immport database. The protein-protein interaction (PPI) network of 87 immune-related DEGs is constructed with STRING database **(E)** and Cytoscape software **(F)**. Color from red to yellow and circular sizes from large to small indicate the betweenness centrality of genes from high to low. Betweenness centrality is estimated by the cytoNCA plugin.

We first performed PCA on the gene expression data, as shown in **Figure 4A**. PCA segregated treated from control mice, suggesting significant differences in gene expression profiles between the two groups. We identified 1059 DEGs in total (**Supplementary Table 3**), among which 757 were significantly downregulated, including IGHD, EBF1, and CR2, while 302 genes were upregulated (**Figure 4B**) in the treated group. The expression levels of DEGs are shown in **Figure 4C**.

To fully understand the biological mechanism of BAFF blockade in SLE, we used multiple external databases to interpret DEGs. We identified key genes encoding cytokines that play important roles in autoimmune disease (30). CCL12, CXCL11, CXCL3, IL-10, IL-1A, IL-21, TNFSF15, and TNFSF18 were downregulated after treatment (**Supplementary Figures 2A, B**). Among these, IL-21 and CXCL13 drive B cell activation, differentiation, and antibody production (31, 32). The expression of CXCL5 (**Supplementary Figures 2A, B**), which suppresses neutrophil and myeloid activation, degranulation, and proliferation, was significantly higher in the treatment group (33). Next, we analyzed ISGs that correlate with SLE disease activity (34). Changes in the expression levels of ISGs were observed in the two groups (**Supplementary Figure 2C**). However, no ISGs meet the criteria for the DEGs, even though the treatment group showed an improvement in disease activity.

Next, we focused on immune-related genes (IRGs). Among these, 70 DEGs were downregulated, and 17 were upregulated after BAFF-R-Fc treatment (**Figure 4D**; **Supplementary Table 4**). To determine the interactions between these immune-related DEGs, a PPI network was constructed using the STRING database (**Figure 4E**). We then evaluated the betweenness centrality of each gene using Cytoscape and arranged the genes based on betweenness centrality (**Figure 4F**). IL-10, CD19, and CD40 were the top three highest-ranking IRGs. The downregulation of IL-10 expression level is consistent with the serum level we detected after treatment (**Supplementary Figure 3B**). Immune complexes can induce IL-10 production from peripheral blood mononuclear cells through the Fcγ receptor, further enhancing autoantibody production and maintaining the hyperactivity of B cells in SLE (35). Further, interactions between the CD40 ligand on T cells and CD40 on B cells lead to the over-activation of T, and B cells in SLE (36).

Finally, we used the TRRUST v2 database to identify key TFs. Among the downregulated DEGs, we observed 58 TFs, 33 of which with a *p*-value < 0.05, suggesting that these might play important roles during treatment (**Supplementary Table 5**). The functions of these TFs, such as PAX5, XBP1, CEBPB, NF-κb1, EBF1, MEF2C, and POU2AF1 were strongly associated with B cell activation and proliferation (**Supplementary Figure 2D**) (37–41), further providing more evidence that BAFF blockade suppresses B cells proliferation in SLE.

### 3.5 Functional and gene set enrichment analysis

**Figure 5:**
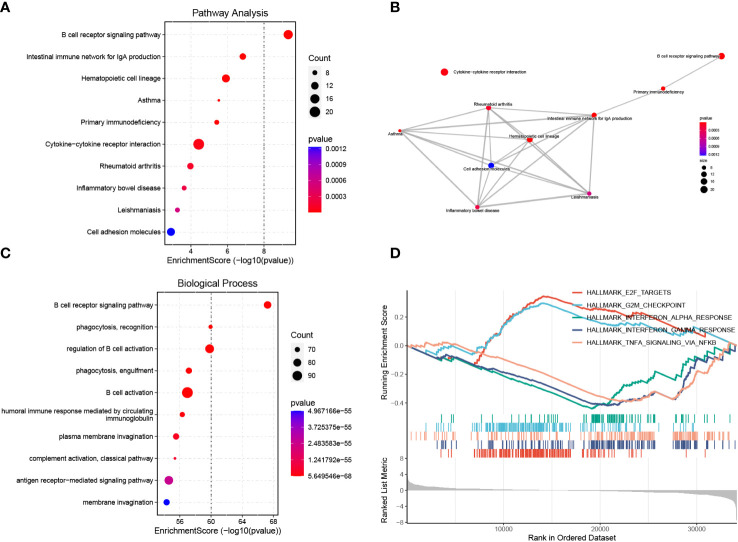
Functional enrichment analysis of DEGs and GSEA. **(A)** Enrichment analysis of DEGs using the KEGG pathway database. The y-axis represents pathway names, the x-axis represents enrichment score in -log10(*p* value). The number of genes enriched on pathways is represented by the plot size, larger sizes indicate more significant DEGs enrichment on pathways. The color gradient ranges from blue to red, reflecting decreasing *p* values from high to low. **(B)** Pathway-pathway interaction network of the ten KEGG pathways with the smallest *p* values is shown. **(C)** GO biological process enrichment analysis of DEGs. **(D)** Activated or inhibited pathways were identified using GESA based on hallmark gene sets downloaded from the MSigDB database. The enrichment score line graph highest peak at the bottom an enrichment score < 0 represents the pathway is inhibited, the highest peak at the top and an enrichment score > 0 represents the pathway is activated. Five pathways with the smallest *p* values were represented by different colors.

We performed KEGG pathway and GO biological process enrichment analyses to reveal the underlying mechanism of BAFF blockade in MRL/lpr mice based on DEGs. KEGG pathway enrichment analysis demonstrated that DEGs were strongly enriched in the BCR signaling pathway, antibody production, cytokine-cytokine receptor interaction, and several autoimmune diseases, as shown in **Figure 5A**. These pathways are closely linked (**Figure 5B**). Enriched GO biological process terms included BCR signaling pathway, membrane invagination, B cell activation, and immune recognition (**Figure 5C**). Considering that some genes with important biological functions were not significantly differentially expressed, we performed GSEA to confirm the enrichment of hallmark pathways using whole gene expression profiles. Noticeably, the IFN-α/γ and TNFα responses were significantly downregulated, consistent with serum levels of IFN-γ and TNFαwe detected in the two groups (**Supplementary Figures 3C, D**). In addition, E2F targets, and G2M checkpoint gene sets, two hallmark gene sets related to cell cycle progression and cell proliferation, were activated in the treatment compared to the control group (**Figure 5D**; **Supplementary Table 6**). These results identify the inhibited signal pathways in MRL/lpr mice that received anti-BAFF therapy.

### 3.6 Immune cell composition of spleen analysis

**Figure 6:**
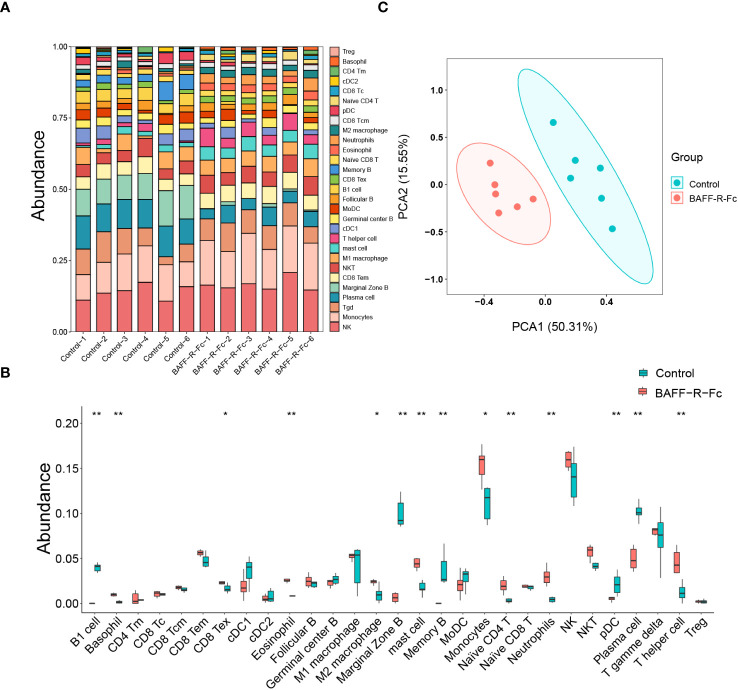
Using gene expression profiles to predict the relative proportion of immune cells. **(A)** The relative abundance of 29 immune cells in the spleen of each sample was estimated by ImmuCellAI-mouse. **(B)** Comparison of each immune cell type in control and BAFF blockade groups. **(C)** Principal components analyses (PCA) of immune cells’ proportions. The first two principal components PCA1 and PCA2 are evaluated for each sample vector and plotted. cDC, conventional dendritic cell. CD8 Tc, CD8 cytotoxic T cell. pDC, plasmacytoid dendritic cell. NKT, natural killer T cell. CD8 Tc, CD8 cytotoxic T cell. T gamma delta, γδ T cell. MoDC, monocyte-derived dendritic cell. NK, natural killer cell. Treg, regulatory T cell. CD8 Tex, exhausted CD8 T cell. CD4 Tm, memory CD4 T cell. CD8 Tcm, CD8 central memory T cell. CD8 Tem, CD8 effector memory T cell. Statistical analysis was performed with Mann-Whitney U-test. Bars represent mean ± S.D. *p < 0.05; **p < 0.01.

Using gene expression profiles, we computed the relative proportion of immune cells in MRL/lpr mice spleens (**Figure 6A**). Distinct changes were observed after BAFF-R-Fc treatment, as shown in **Figure 6B**. Significant decreases in the proportion of B cell subsets, including memory B, B1, plasma, and marginal zone B cells, as well as plasmacytoid dendritic cells (pDCs) were observed. Meanwhile, the proportion of monocytes, basophils, eosinophils, mast cells, neutrophils, M2 macrophages, naïve CD4 T cells, T helper cells, and exhausted CD8 T cells significantly increased. Principal component analysis (PCA) revealed significant group-bias clustering in the proportion of immune cells in samples from treated mice and controls (**Figure 6C**). The decrease in B cell subpopulations was consistent with the downregulation of TFs related to B cell proliferation.

### 3.7 The correlations between immunoglobulins, B cell proportion, BCR repertoire diversity, and richness

**Figure 7:**
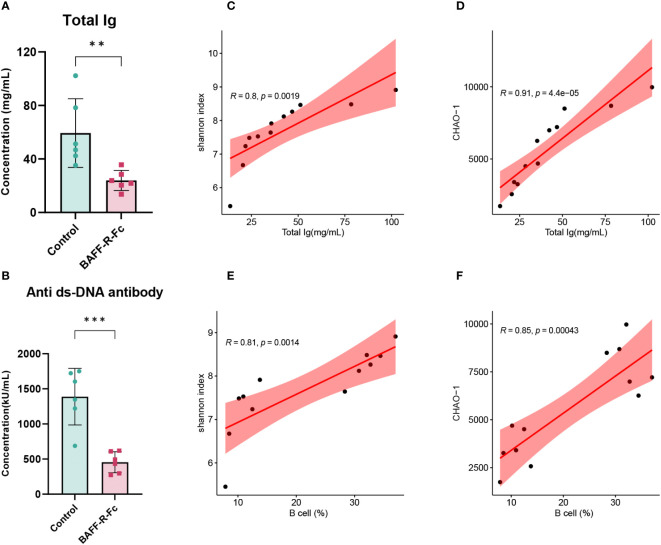
Correlation analysis of the BCR diversity, richness, immunoglobulins levels, and B cells proportion. **(A)** Serum levels of total immunoglobulins in the two groups. **(B)** Anti-dsDNA antibodies titers of BAFF blockade and control mice. Titers were shown in activity units. Correlation between total immunoglobulins levels and the Shannon index **(C)** and CHAO-1 **(D)**. Correlation between B cells proportion and the Shannon index **(E)** and CHAO-1 **(F)**. Pearson correlation coefficient was performed to indicate the extent of linear correlation between two arbitrary variables. Confidence intervals and line of regression were shown. Statistical analysis was performed with unpaired t-test comparing untreated to treated. Bars represent mean ± S.D. **p < 0.01; ***p < 0.001.

To investigate the correlation between BCR diversity, richness, B cell proportion in the spleen, and serum immunoglobulins levels, we conducted a correlation analysis of these variables in the two groups. First, we measured the serum levels of immunoglobulins. All immunoglobulin isotypes, including IgA, IgM, IgG1, IgG2a, IgG2b, and IgG3, exhibited lower serum levels following treatment (**Figure 7A**; **Supplementary Figure 3A**). We also measured anti-dsDNA antibody titers in MRL/lpr mice, which are highly specific to SLE and positively correlated with disease activity. As expected, BAFF-R-Fc decreased anti-dsDNA antibody titers compared to that in the control (**Figure 7B**). Correlation analysis revealed significant positive correlations between immunoglobulin levels and BCR diversity and richness **(Figures 7C, D**). Besides, B cell proportion was also positively correlated with BCR diversity and richness (**Figures 7E, F**). These data suggest that anti-BAFF therapy in MRL/lpr mice could impact the BCR diversity and richness by depleting B cells and preventing antibody production, which is relevant to prognostic outcomes.

### 3.8 BAFF-R-Fc regulated splenic B cell subset development and reduced ASC numbers

**Figure 8:**
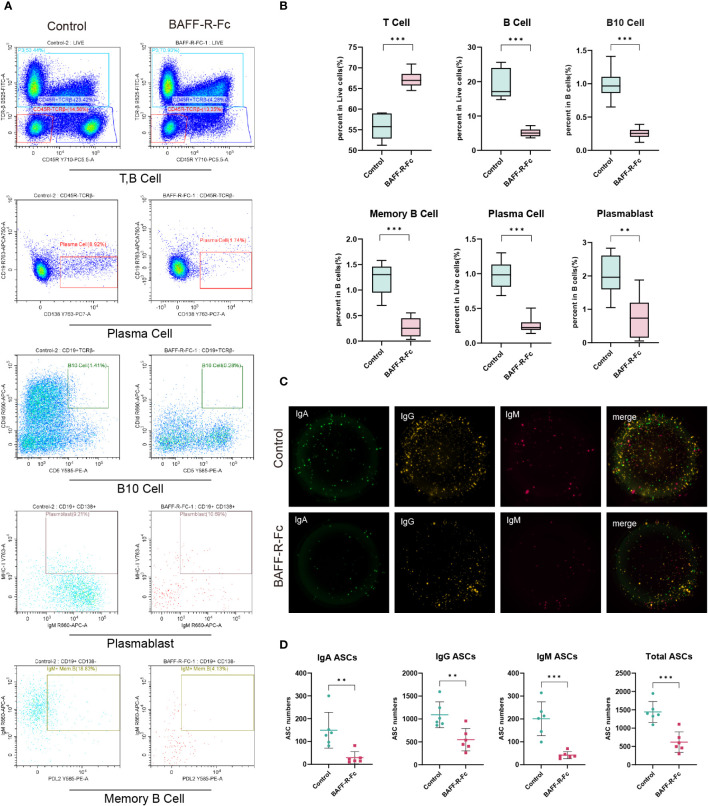
BAFF blockade regulated splenic T cells, B cell subsets, and ASCs. B cell subsets and T cells were obtained from MRL/lpr mice spleens, and detected by flow cytometry. IgM-, IgA-, and IgG-secreting cells were detected using the FluoroSpot assay. **(A)** Representative FACS analysis of T cells (TCRβ^+^), B cells (TCRβ^-^CD45R^+^), and B cell subsets including plasma cells (CD19^-^CD138^+^) plasmablast (IgM^+^MHCII^+^CD138^+^), B10 cells (CD1d^+^CD5^+^) and memory B cells (PD-L2^+^IgM^+^MHCII^+^). **(B)** The box plot shows the percent of B cells, T cells, and plasma cells in splenic lymphocytes and the percent of memory B cells plasmablasts, and B10 cells in B cells. **(C)** Images of BAFF blockade and control’s IgA/IgM/IgG FluoroSpot with three-color spots: IgA-green spot, IgM-red spots, IgG-yellow spot, and composite picture of three-color spots. **(D)** Number of IgA, IgM, IgG, and total ASCs spots in 5×10^4^ cells detected by FluoroSpot assay. Statistical analysis was performed with unpaired t-test and Holm-Bonferroni correction comparing untreated to treated. Bars represent mean ± S.D. **p < 0.01; ***p < 0.001.

We validated B cell depletion caused by BAFF blockade by flow cytometry and FluoroSpot. We first determined the proportion of B cells (TCRβ^-^CD45R^+^), which was obviously reduced in the spleen of mice treated with BAFF-R-Fc (**Figures 8A, B**). Consistently, memory B cells (PD-L2^+^IgM^+^MHCII^+^) and B10 cells (CD1d^+^CD5^+^), a regulatory B cell subset producing IL10 to control T cell-dependent inflammatory responses (42), were also reduced (**Figures 8A, B**). Meanwhile, we found that the proportion of T cells (TCRβ^+^) was higher than that in controls (**Figures 8A, B**). We confirmed that BAFF-R-Fc suppressed autoantibody production by decreasing the number of ASCs, including plasma cell precursors (plasmablasts, IgM^+^MHCII^+^CD138^+^) and plasma cells (CD19^-^CD138^+^) (**Figures 8A, B**). We then investigated whether BAFF blockade only leads to the depletion of certain ASCs, subjecting them to FluoroSpot analysis. The numbers of IgM-, IgA-, and IgG-secreting cells were consistently reduced (**Figures 8C, D**). These immune cell proportions obtained by flow cytometry were consistent with the predictions using ImmuCellAI-mouse, with Pearson’s correlation coefficients ranged from 0.66 to 0.89 (**Supplementary Figures 3E–H**). Our results suggest that BAFF blockade contributes to the depletion of these B cell subsets.

## 4 Discussion

In this study, we performed transcriptome and BCR repertoire analyses on the spleens of MRL/lpr mice treated with the BAFF-F-R-Fc fusion protein. We noted lower BCR diversity, richness, and a decrease in the number of unique clonotypes in treated mice. IGHV gene usage was also different from that in controls. Additionally, our study successfully detected changes in the proportions of splenic lymphocyte subpopulations and gene expression. We observed downregulation of IFN-α/γ and TNF-α responses, with most DEGs related to B cell proliferation and antibody production. These results imply that BAFF blockade dramatically remodeled the immune microenvironment in the spleen of MRL/lpr mice and regulated the B cell receptor repertoire.

The comprehensive characterization of the BCR repertoire has improved our knowledge of autoimmune disease pathogenesis (43, 44). The presence of abnormal specific clonotype expansion and diversity as well as the abnormal usage of IGHV genes in SLE indicate a broad dysregulation of the BCR repertoire (45, 46). Thus, we studied the effect of BAFF blockade on the BCR repertoire. Tipton et al. previously noted that the plasma and plasmablast populations of patients with SLE expand considerably as a result of prolonged immune activation, which resulting in an increase in the proportion of hyperexpanded clonotypes and a decrease in BCR repertoire diversity (8). Interestingly, instead of returning to normal levels, a further decrease in BCR repertoire diversity and richness were observed in the BAFF-R-Fc treatment group compare to the control. Besides, hyperexpanded clonotypes occupied more BCR repertoire space in the treatment group. So, we hypothesized that BAFF blockade reduces the number of plasma cells and plasmablasts, while also sharply reducing the number of other B cell subpopulations, which causes dramatic declines in BCR repertoire diversity and richness. As we found the BCR diversity and richness were positively correlated with B cells proportion. Similar outcomes have been reported in patients receiving stable immunosuppressive therapy such as mycophenolate mofetil and leflunomide (47).

Huang et al. reported a notable loss of IGHV4-34 among plasmablasts and a mutated IgM BCR repertoire in chronic belimumab-treated individuals when compared to controls (48). Here, we found no significant difference in the usage of IGHV genes after BAFF-R-Fc therapy in MRL/lpr mice. Notably, some VH genes such as VH1-14, VH1S61, and VH1-82 showed extremely high frequencies in both treated and control mice. Further studies are required to confirm whether these genes are involved in the production of autoreactive antibodies. Nonetheless, we observed a difference in the frequency distribution of VH genes between the treated mice and the controls, resulting in larger Jensen-Shannon divergence between them. We noticed that our analysis of variations in IGHV gene usage successfully distinguished between the treatment and control groups.

Wardemann and Mefre previously showed that a longer immunoglobulin heavy chain CDR3 correlates with antibody autoreactivity in SLE (49, 50). We found that no significant difference in CDR3 mean length of IGHV in BAFF-R-Fc-treated mice compared to those observed in controls, consistent with a previous study (47). However, the distribution of CDR3 lengths was altered, with the most commonly used length decreasing from 15 amino acids to 14 amino acids.

Here, we systematically analyzed the transcriptome profiles of spleens from MRL/lpr mice and identified genes regulated by anti-BAFF therapy. Similar to a previous study on gene expression changes in patients treated with tabalumab (51), most of the downregulated genes were associated with B cell proliferation and the B cell receptor signaling pathway, confirming the cell type-specific effects of BAFF. Consistent with the decrease in B cell subsets we observed in the spleens of treated mice using flow cytometry, a similar decline in B cell subsets was observed in patients with SLE treated with belimumab (52).

Elevated ISGs expression in the peripheral blood cells of patients with SLE is associated with disease activity (53, 54). However, in two phase III trials of BAFF blockade with tabalumab, patients with improved disease activity showed stable IFN response gene expression (51). We analyzed downregulated DEGs and confirmed that no significant changes in the expression levels of ISGs in MRL/lpr mice treated with BAFF-R-Fc compared to the control. We then performed GSEA to identify the integrated expression levels of the IFN signature. The GSEA results successfully demonstrated a significant downregulation of IFN-α/γ and TNF-α responses after anti-BAFF therapy, consistent with the observed decrease in IFN-γ and TNF-α serum levels.

In our study, we detected a decrease in splenic IL-10 mRNA levels and serum IL-10 protein levels following BAFF blockade. The PPI network of IRGs suggested that IL-10 is involved in the regulation of B cell proliferation. While the role of IL-10 in SLE is controversial, significantly increased serum levels of IL-10 have been reported in patients, being positively correlated with SLE disease activity and dsDNA antibody titers (55, 56). In the early stages of disease, IL-10 inhibits IFN-γ-mediated autoantibody production and renal inflammation. However, in the later stages, excess IL-10 enhances major histocompatibility complex II expression on B cells and their differentiation into plasmablasts that secrete IgM and IgG (57–59). Thus, the downregulation of IL-10 expression level may contribute to mitigating disease activity.

The disruption of immune tolerance mechanisms leads to the expansion of B cell subpopulations and is a characteristic of SLE. Herein, BAFF blockade reduced the number of B cell subpopulations, including plasma cells, plasmablasts, and marginal zone B cells, in the spleen, which is consistent with previous studies (60, 61). We examined whether blocking BAFF resulted in the preferential reduction of certain plasma cell subpopulations. Our findings revealed significant reductions in the numbers of IgM, IgA, and IgG ASCs, consistent with the decreased serum immunoglobulin isotype levels. Our results noted that anti-BAFF therapy depleted memory B cells in MRL/lpr mice, in contrast to a previous study where patients treated with belimumab retained memory B cells (61). One possible reason is that the depletion of B cells occurs in the early phase of the disease in mice. Germinal center B cells were preserved following anti-BAFF therapy, in accordance with observations in patients receiving long-term belimumab treatment (60). In the absence of BAFF, germinal center responses were reportedly attenuated, yet still allowed antibody production (62). The depletion of CD1D^+^CD5^+^ B10 Bregs was also observed after BAFF blockade. Iwata et al. reported a significant increase in blood B10 cell frequency among patients and mice with autoimmune diseases compared to healthy controls (42, 63). The survival of CD19+-/- NZB/W mice greatly improved following the adoptive transfer of B10 cells (64), with these cells suppressing the generation of IFN-γ, TNF-α, and pathogenic autoantibodies in MRL/lpr mice, independently from IL-10 (65). The above-described findings raise the question of whether preserving the B10 subpopulation while blocking BAFF to deplete B cells will achieve better therapeutic outcomes.

Early studies reported that pDCs were chronically activated and continuously released IFN-α in patients with SLE (66, 67). DNA-containing immune complexes from SLE patient sera promote the synthesis of chemokines and cytokines by pDCs through cooperative interactions between CD32 and Toll-like receptor 9 (68, 69). Here, we observed a decrease in the proportion of pDCs and the downregulation of IFN-α response in MRL/lpr mice after BAFF blockade. However, more experimental research is needed to fully understand the mechanism.

Our study had several limitations. First, we only obtained the bulk transcriptome and BCR repertoire. Thus, we were unable to examine gene expression levels in different immune cell types and determine interactions between immune cells. Second, the lack of databases with information on immune receptors in mice prevented us from annotating our data and identifying autoimmunity-associated receptors. We could not confirm whether the highly expanded clonotypes were related to autoantibody. Third, the prediction of pDC and other myeloid cells by cell deconvolution algorithms was not accurate enough (70, 71), and required experimental verification. Finally, a large-scale clinical trial is required to confirm the potential of BCR sequencing in predicting disease prognosis.

## Data availability statement

The RNA-seq and BCR repertoire data during the current study are available in the SRA repository, accession: PRJNA1017616.

## Ethics statement

The animal study was approved by Animal Ethics Committee of Tongji University (No. TJLAC-018-032). The study was conducted in accordance with the local legislation and institutional requirements.

## Author contributions

TH: Conceptualization, Data curation, Formal Analysis, Writing – original draft. CP: Methodology, Writing – original draft. XX: Formal Analysis, Writing – original draft. YF: Methodology, Writing – original draft. JZ: Validation, Writing – original draft. HG: Funding acquisition, Supervision, Writing – review & editing. JF: Conceptualization, Funding acquisition, Project administration, Resources, Supervision, Writing – review & editing.
