# Supplementary material for: Effect of BAFF blockade on the B cell receptor repertoire and transcriptome in a mouse model of systemic lupus erythematosus
Source: Front Immunol. 2024 Jan 9;14:1307392. doi: 10.3389/fimmu.2023.1307392 (PMC10803406; doi:10.3389/fimmu.2023.1307392)
Supplement: Supplementary file 1 [file DataSheet_1.docx]

Supplementary Material

# Supplementary Figures and Tables

## Supplementary Tables

**Table S1**: The usage of IGHV genes in B cell receptor repertoire of MRL/lpr mice.

**Table S2**: Public complementarity-determining region 3 amino acid (aa) sequences shared by at least 8 samples.

**Table S3**: A list of differentially expressed genes (DEGs).

**Table S4**: A list of immune-related differential expressed genes (IRGs).

**Table S5**: Transcriptional factors （TFs） with *p*-value < 0.05 in down-regulated DEGs.

**Table S6**: Results of gene set enrichment analysis with HALLMARK gene sets.

## Supplementary Figures


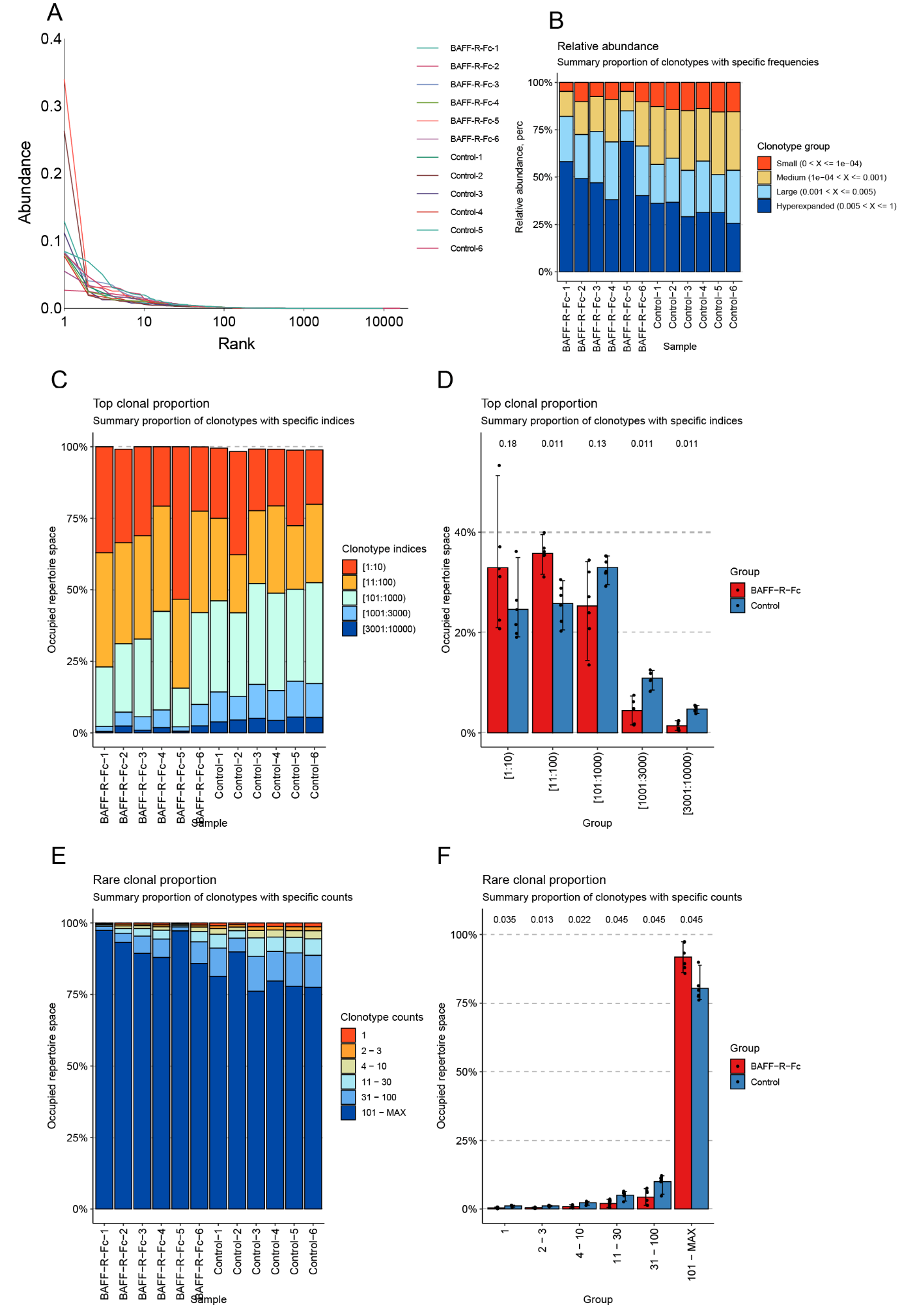


**Supplementary Figure 1. (A)** Rank-abundance curve of each sample. The horizontal coordinate represents the ranking of clonotypes according to their relative abundance from largest to smallest, the vertical coordinate represents the relative abundance of each clonotype. **(B)** The cumulative relative abundance of small-expanded (0-0.01%), medium-expanded (0.01%-0.1%), large-expanded (0.1%-0.5%), and hyper-expanded (0.5%-100%) clonotypes in two groups. **(C, D)** The cumulative relative abundance of top 1-10, 11-100, 101-1000, 1001-3000, and 3001-10000 clonotypes in the two groups. **(E, F)** The cumulative relative abundance of clonotypes count 1, 2-3, 4-10, 11-30, 30-100, and 101-max in the two groups.


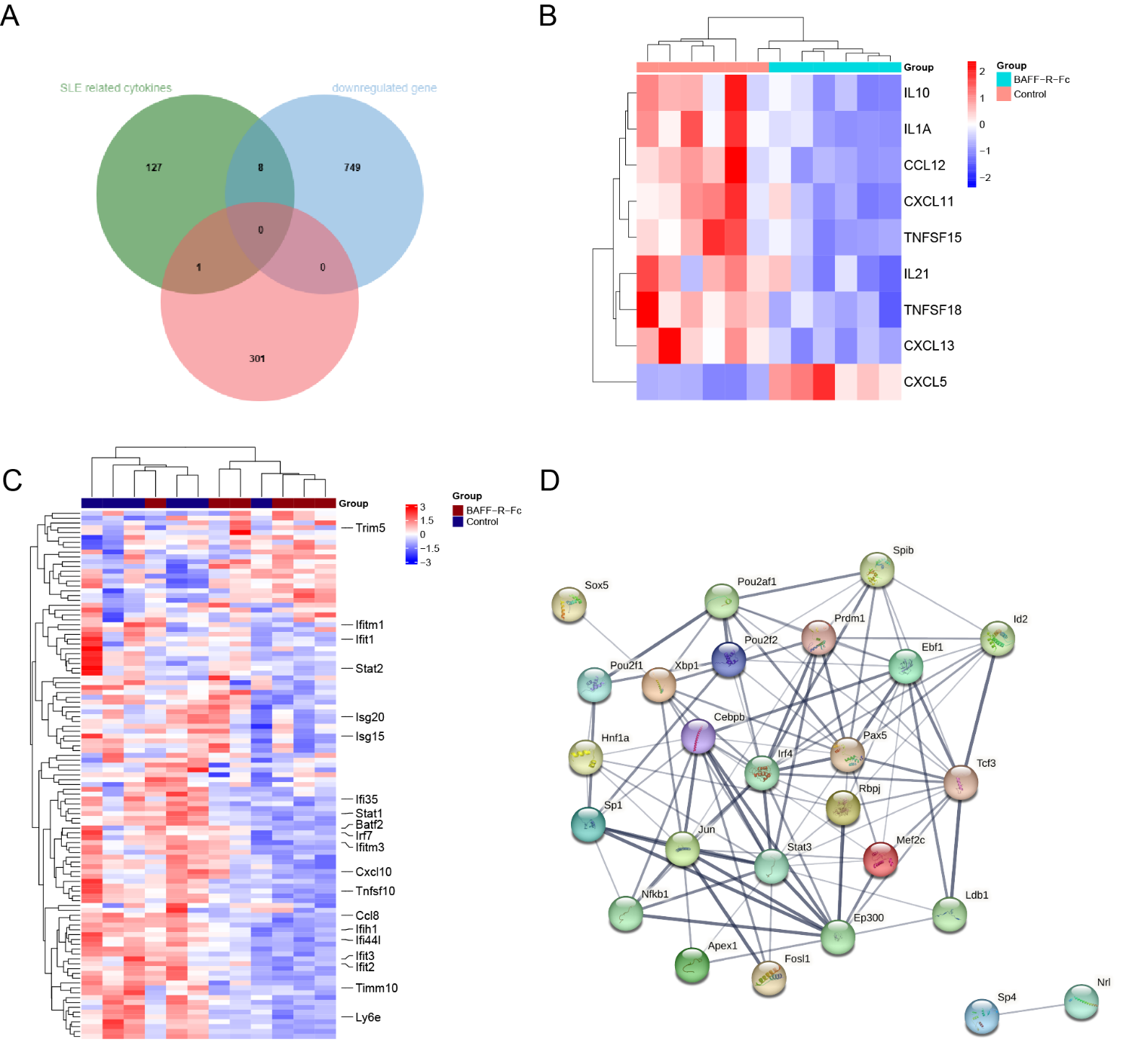


**Supplementary Figure 2.** **(A)** Venn diagram of the overlap among SLE related cytokines and upregulated/downregulated DEGs. **(B)** Heatmap of the expression levels of CCL12, CXCL11, CXCL3, IL-10, IL-1A, IL-21, TNFSF15, CXCL5, and TNFSF18. **(C)** Heatmap of the expression levels of ISGs. **(D)** The protein-protein interaction (PPI) network of 33 TFs.

**
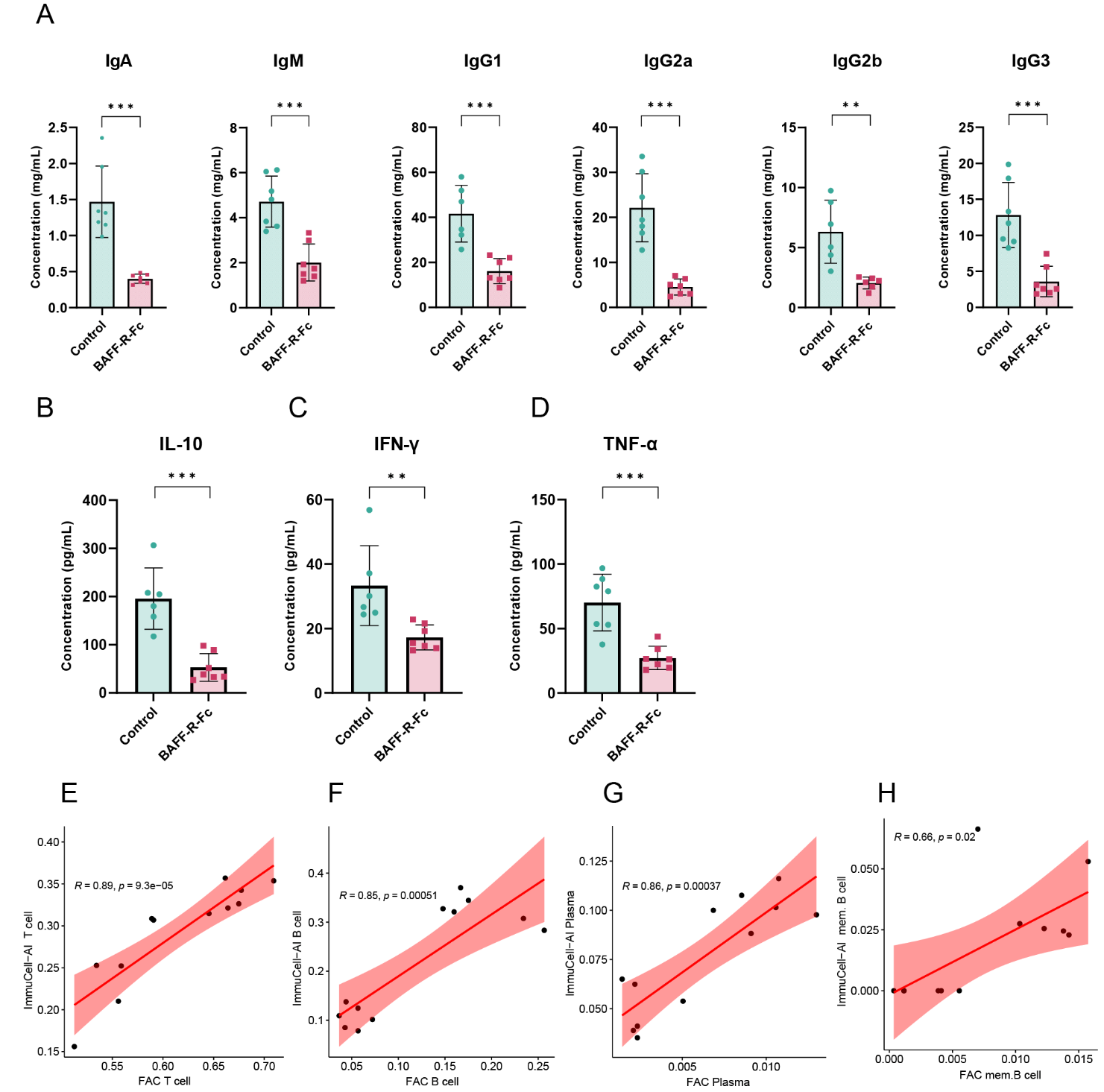
**

**Supplementary Figure 3.** **(A)** Serum levels of immunoglobulins isotypes (IgA, IgM, IgG1, IgG2a, IgG2b, IgG3) in the two groups. Serum levels of IL-10 **(B)**, IFN-γ **(C)**, and TNF-α **(D)** in the two groups. Pearson’s correlation between T cell **(E)**, B cell **(F)**, plasma cell **(G)**, and memory cell **(H)** proportions obtained by ImmuCellAI-mouse and flow cytometry was used to assess the performance of ImmuCellAI-mouse.
